# Impact of diagnostic errors on adverse outcomes: learning from emergency department revisits with repeat CT or MRI

**DOI:** 10.1186/s13244-021-01108-0

**Published:** 2021-11-03

**Authors:** Yura Ahn, Gil-Sun Hong, Kye Jin Park, Choong Wook Lee, Ju Hee Lee, Seon-Ok Kim

**Affiliations:** 1 Department of Radiology and Research Institute of Radiology, Asan Medical Center, University of Ulsan College of Medicine, 88 Olympic-ro 43-gil, Songpa-gu, Seoul, 05505 Republic of Korea; 2 Department of Clinical Epidemiology and Biostatistics, Asan Medical Center, Seoul, Republic of Korea

**Keywords:** Emergency health services, Diagnostic imaging, Diagnostic errors, Quality indicators, Adverse outcomes

## Abstract

**Background:**

To investigate diagnostic errors and their association with adverse outcomes (AOs) during patient revisits with repeat imaging (RVRIs) in the emergency department (ED).

**Results:**

Diagnostic errors stemming from index imaging studies and AOs within 30 days in 1054 RVRIs (≤ 7 days) from 2005 to 2015 were retrospectively analyzed according to revisit timing (early [≤ 72 h] or late [> 72 h to 7 days] RVRIs). Risk factors for AOs were assessed using multivariable logistic analysis. The AO rate in the diagnostic error group was significantly higher than that in the non-error group (33.3% [77 of 231] vs. 14.8% [122 of 823], *p* < .001). The AO rate was the highest in early revisits within 72 h if diagnostic errors occurred (36.2%, 54 of 149). The most common diseases associated with diagnostic errors were digestive diseases in the radiologic misdiagnosis category (47.5%, 28 of 59) and neurologic diseases in the delayed radiology reporting time (46.8%, 29 of 62) and clinician error (27.3%, 30 of 110) categories. In the matched set of the AO and non-AO groups, multivariable logistic regression analysis revealed that the following diagnostic errors contributed to AO occurrence: radiologic error (odds ratio [OR] 3.56; *p* < .001) in total RVRIs, radiologic error (OR 3.70; *p* = .001) and clinician error (OR 4.82; *p* = .03) in early RVRIs, and radiologic error (OR 3.36; *p* = .02) in late RVRIs.

**Conclusion:**

Diagnostic errors in index imaging studies are strongly associated with high AO rates in RVRIs in the ED.

**Supplementary Information:**

The online version contains supplementary material available at 10.1186/s13244-021-01108-0.

## Key points

- Diagnostic errors in index emergency imaging are strongly associated with adverse outcomes.
- Diagnostic errors most frequently occur in neurologic and digestive diseases.
- Adverse outcomes are highest at early emergency revisits if diagnostic errors occur.
- Revisits with repeat imaging could be a quality indicator for emergency radiology.

## Background

Several studies have investigated the incidence and causes of medical errors [1–5]. However, such analyses remain challenging owing to the lack of effective methods for measuring medical errors, limited sources of reliable data, and difficulties in detecting errors in clinical practice settings. In emergency medicine, unplanned revisits to the emergency department (ED) are known care quality indicators and have been proposed as potential triggers of diagnosis or signifiers of medical errors [6–9]. Triggers are clinical outcomes indicating the possibility of adverse outcomes (AOs). Screening for triggers can identify problem cases that require further investigation [2, 10–12]. It has been reported that up to 6.8% of patients revisit the ED within 7 days of discharge from the ED [6]. An association likely exists between medical errors and poor outcomes among patients who make unplanned revisits to the ED [13].

Similarly, relatively little information exists about diagnostic errors in emergency radiology. An effective method for measuring diagnostic errors in emergency radiology is required. Most published studies on radiologic errors have been random case reviews, medical malpractice claim analyses, insurance record analyses, or analyses of repeat imaging examinations [14–22]. However, as such studies reported selected cases by clinicians or were based on the discrepancy between long-term follow-up images, they have limited ability to reflect diagnostic errors in the emergency radiology service. Moreover, those studies were focused on error classifications in radiology but did not address the association between diagnostic errors and clinical outcomes. Given the increasing use of computed tomography (CT) and magnetic resonance imaging (MRI) in the ED and their impact on the decision making of clinicians, this issue is expected to be particularly important in the context of emergency care [23]. We hypothesized that the diagnostic errors from the index imaging led to the patients revisit with repeat imaging (RVRIs) at the ED, resulting in AOs (delayed operations, 30-day in-hospital mortality, intensive care unit [ICU] admission). We aimed to analyze whether diagnostic errors occurred in the index imaging and their impact on the AOs in the RVRIs at the ED.

## Materials and methods

### Study sample

**Fig. 1:**
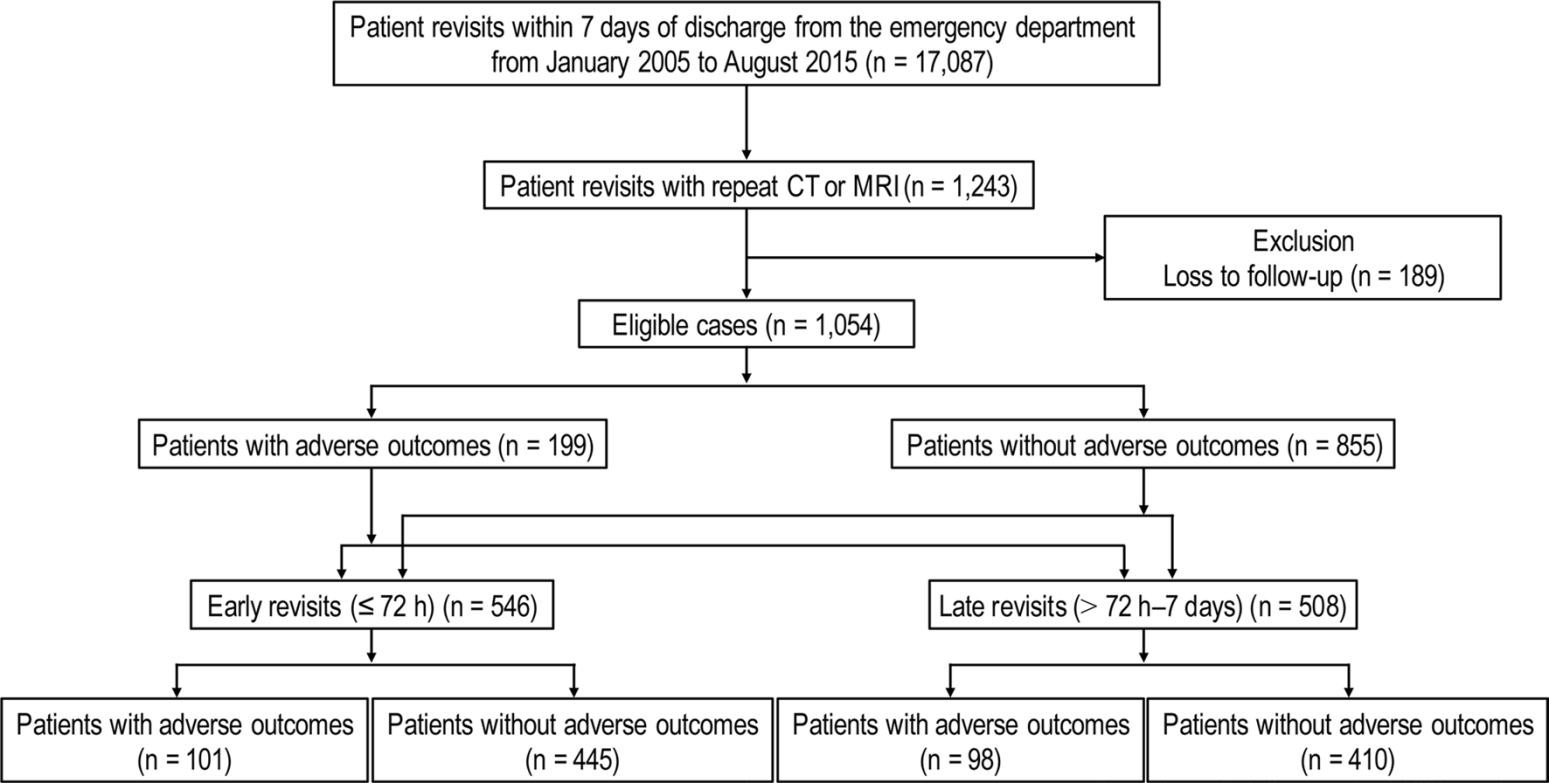
Flowchart of study enrollment

This retrospective study was conducted at a tertiary academic hospital that manages > 150,000 ED cases per year. This study included RVRIs from January 2005 to August 2015. Patient revisits were defined as a visit to the same ED within 7 days of discharge from the ED [6, 24]. Repeat CT or MRI was defined as one that followed a preceding CT or MRI regardless of the scanning site among the patient revisit cohorts at the ED. The inclusion criteria were as follows: (1) age ≥ 18 years, (2) revisits within 7 days after ED discharge, (3) repeat CTs or MRIs, and (4) admission to the hospital or a subsequent outpatient follow-up clinic visit after the ED revisit (patients were included if they were followed up [> 30 days] in an outpatient clinic or if an AO occurred [≤ 30 days]). The exclusion criterion was loss to follow-up. The outcome variable (presence or absence of AOs) was investigated in terms of diagnostic accuracy and disease category at the index ED visit, as well as in terms of RVRI timing (early RVRIs [≤ 72 h] vs. late RVRIs [> 72 h to 7 days]), based on parameters set by previous studies [9, 10, 13, 25] (Fig. 1; see Additional file 1-1 for the emergency radiology service system).

### Data collection

At the index ED visit, the collected data were age, sex, comorbidities, initial radiologic reports, radiology reporting time (RT), clinician’s diagnostic decision, and clinician’s decision time. As preliminary reporting is more commonly used for the clinical decision than final reporting in the ED, the RT was determined based on the time when the preliminary written report was prepared. At the ED revisit and follow-up period, the collected data were radiologic reports, clinical diagnosis, and AOs. The disease categories (determined by comparisons between index visits and revisits and by diagnosis during follow-up) in the index visit were classified according to modified guidelines from the 11th edition of the International Classification of Diseases [26]. AOs were defined as any sub-optimal outcomes (delayed operations, 30-day in-hospital mortality, or ICU admission) experienced by patients during ED consultations or within 30 days [27]. In addition to conventional AOs, we included delayed operations as they are related to in-hospital mortality, longer length of stay, and higher cost [28]. Furthermore, there is an interrelationship between radiology reporting time and surgeon’s decision-making timing [29].

### Analysis of diagnostic errors stemming from index imaging studies

The nature of patient revisits with repeat imaging was categorized into three groups: 1) radiologic error (misdiagnosis or delayed RT [D-RT]), 2) clinician error (inappropriate patient triage or failure to order the appropriate imaging examinations), and 3) non-error (patient- or illness-related factor: progressed diseases or new symptoms or diseases) according to a modified classification with reference to a previous study [30] (see Additional file 1-2 for details in the analysis of diagnostic errors). In brief, to investigate radiologic errors, three radiologists (GS Hong, KJ Park, and Y Ahn, with > 14, > 8, and 4 years of experience in CTs and MRIs, respectively) reviewed the radiology reports of index and repeat imaging studies. When there were discrepancies on critical findings between two radiology reports of the index and repeat imaging examinations, two board-certified radiologists (GS Hong and KJ Park) reviewed the images to identify whether or not the discrepancies were due to a misdiagnosis. The consensus about radiologic errors was reached through a discussion. When the RT was later than the clinician’s decision time, index images were also reviewed by two radiologists (GS Hong and KJ Park) and cases with critical positive findings on the index scans were considered D-RT. Clinician errors comprised inappropriate patient triage (i.e., underestimation of the patient’s condition and scanning at inappropriate sites) and failure to order appropriate imaging examinations (i.e., the ordered imaging studies were unsuitable for diagnosing the patient’s disease). However, detecting positive findings on an index scan regardless of clinician error (e.g., unreported acute cholecystitis present on the index chest CT scan) was considered a radiologic misdiagnosis. The cause and preventability of diagnostic errors were also analyzed.

### Statistical analysis

Comparisons between the study groups with and without AOs were performed using the Chi-square test and Fisher’s exact test for categorical variables and Student’s *t* test and the Mann–Whitney *U* test for continuous variables. Univariable and multivariable logistic analyses were performed with backward elimination using penalized maximum likelihood estimation to identify independent risk factors associated with AOs according to RVRI timing. To adjust for potential confounding factors, we performed 1:1 matching using a greedy algorithm, in which randomly selected individuals in the patient factor group were paired with comparable individuals in the radiologic error and clinician error groups who fulfilled the following matching criteria: age (± 5 years), sex, revisit timing, comorbidity (malignancy), and six disease categories (neurologic, digestive, neoplasm, respiratory, circulatory, and other diseases). In the matched set, the risk of AOs was compared using a logistic model with generalized estimating equations that accounted for the clustering of matched pairs. All statistical analyses were performed using SPSS Statistics for Windows (version 21, IBM Corp.) and SAS (version 9.4, SAS Institute). A two-sided *p* values < 0.05 were considered to indicate statistical significance.

## Results

### Patient characteristics

Table 1 summarizes the patient characteristics. During the study period, 7.3% (1243 of 17,087) of ED revisits involved repeat CT or MRI in the ED. The overall median follow-up duration for all eligible patients was 28 days (interquartile range, 13–39 days). Among 1054 eligible patients (mean age ± standard deviation, 56.0 ± 15.7 years; 532 men [50.5%]), 18.9% (199 of 1054) experienced AOs. In terms of comorbidities, malignancies (34.6%, 365 of 1054) were the most common. About half (51.8%, 546 of 1054) of the study sample had early RVRIs, and the rest (48.2%, 508 of 1054) had late RVRIs. In total RVRIs, the diagnostic error rate at the index visit was 21.9% (231 of 1054), with radiologic errors contributing to approximately half of the cases (11.5%, 121 of 1054). In total RVRIs, the most common disease categories were neurologic diseases (29.3%, 309 of 1054), followed by digestive diseases (15.1%, 159 of 1054), neoplasms (14.2%, 150 of 1054), and traumatic injuries (9.4%, 99 of 1054). In terms of patient disposition at the revisits, more than half (54.5%, 574 of 1054) of the study sample was admitted or transferred for further treatment, or died in the ED. Among AOs, delayed operation (9.8%, 103 of 1054) was the most common, followed by 30-day in-hospital mortality (6.5%, 69 of 1054) and ICU admission (5.8%, 61 of 1054).

**Table 1:** Baseline characteristics of patients according to the presence or absence of adverse outcomes

Characteristics	All patients	AO group	Non-AO group	*P*
No. of patients	1054	199 (18.9%)	855 (81.1%)	
Age (years)^a^	56.0 ± 15.7	57.9 ± 14.6	55.5 ± 15.9	.05
Sex, male^b^	532 (50.5%)	112 (56.3%)	420 (49.1%)	.07
*Comorbidities*^b^				
Malignancy	365 (34.6%)	96 (48.2%)	269 (31.5%)	< .001
DM	203 (9.3%)	38 (19.1%)	165 (19.3%)	.95
CAD	95 (9.0%)	22 (11.1%)	73 (8.5%)	.26
CKD	54 (5.1%)	8 (4.0%)	46 (5.4%)	.43
Asthma	30 (2.8%)	3 (1.5%)	27 (3.2%)	.34
COPD	25 (2.4%)	8 (4.0%)	17 (2.0%)	.09
*Revisit timing*^b^				.74
Early (≤ 72 h)	546 (51.8%)	101 (50.8%)	445 (52.0%)	
Late (72 h–7 days)	508 (48.2%)	98 (49.2%)	410 (48.0%)	
*Diagnostic error status at index visit*^b^				< .001
1. Radiologic errors	121 (11.5%)	51 (25.6%)	70 (8.2%)	< .001
Misdiagnoses	59 (5.6%)	31 (15.6%)	28 (3.3%)	< .001
D-RT	62 (5.9%)	20 (10.1%)	42 (4.9%)	.006
2. Clinician errors	110 (10.4%)	26 (13.1%)	84 (9.8%)	.18
3. Non-errors	823 (78.1%)	122 (61.3%)	701 (82.0%)	< .001
*Disease categories at index visit*^b^				< .001
1. Neurologic diseases	309 (29.3%)	44 (22.1%)	265 (31.0%)	.01
2. Digestive diseases	159 (15.1%)	45 (22.6%)	114 (13.3%)	.001
3. Neoplasms	150 (14.2%)	51 (25.6%)	99 (11.6%)	< .001
4. Traumatic injuries	99 (9.4%)	6 (3.0%)	93 (10.9%)	< .001
5. Respiratory diseases	50 (4.7%)	10 (5.0%)	40 (4.7%)	.84
6. Genitourinary diseases	42 (4.0%)	5 (2.5%)	37 (4.3%)	.24
7. Infection	38 (3.6%)	5 (2.5%)	33 (3.9%)	.36
8. Circulatory diseases	29 (2.8%)	13 (6.5%)	16 (1.9%)	< .001
9. Healthcare-related complications	17 (1.6%)	6 (3.0%)	11 (1.3%)	.08
10. Other diseases	161 (15.3%)	14 (7.0%)	147 (17.2%)	< .001
(1) Mental or behavioral disorder	27 (2.6%)	2 (1.0%)	25 (2.9%)	.14
(2) Musculoskeletal or connective tissue diseases	15 (1.4%)	5 (2.5%)	10 (1.2%)	.15
(3) Visual and ear diseases	15 (1.4%)	2 (1.0%)	13 (1.5%)	.75
(4) Endocrine or metabolic diseases	12 (1.1%)	2 (1.0%)	10 (1.2%)	1.0
(5) Hematologic diseases	2 (0.2%)	0 (0.0%)	2 (0.2%)	1.0
(6) Immune system diseases	2 (0.2%)	1 (0.5%)	1 (0.1%)	.34
(7) Skin diseases	2 (0.2%)	0 (0.0%)	2 (0.2%)	1.0
(8) Non-diagnostic conditions	86 (8.2%)	2 (1.0%)	84 (9.8%)	< .001
*Patient disposition at revisit*^b^				< .001
Admission	530 (50.3%)	171 (85.9%)	359 (42.0%)	< .001
Discharge	480 (45.5%)	21 (10.6%)	459 (53.7%)	< .001
Transfer	42 (4.0%)	5 (2.5%)	37 (4.3%)	.24
Death in the ED	2 (0.2%)	2 (1.0%)	0 (0.0%)	.04
*Conditions of adverse outcomes*^b^				
Delayed operation	103 (9.8%)	103 (51.8%)	N/A	
30-day in-hospital mortality	69 (6.5%)	69 (34.7%)	N/A	
ICU admission	61 (5.8%)	61 (30.7%)	N/A	

^a^Data are mean ± SD

^b^Data are number of patients, with percentages in parentheses. The sum of percentages may not be exactly 100% owing to rounding

CAD, Coronary artery disease; CKD, chronic kidney disease; COPD, chronic obstructive pulmonary disease; DM, diabetes mellitus; AO, adverse outcome; ED, emergency department; D-RT, delayed radiology reporting time; N/A, not applicable; ICU, intensive care unit

### Disease categories associated with diagnostic errors

**Fig. 2:**
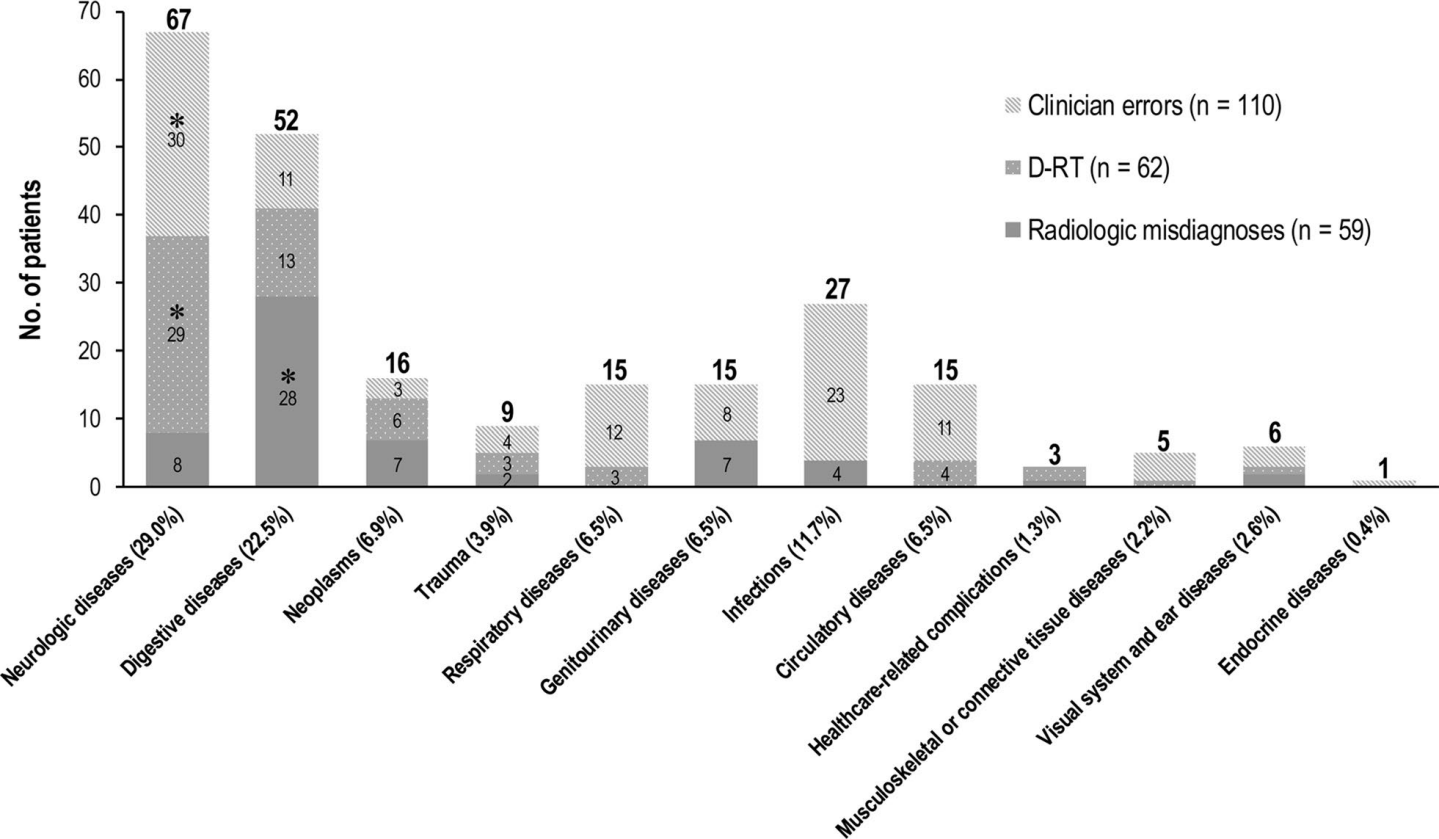
Disease categories according to diagnostic error categories. *The most common disease categories associated with each diagnostic error item. D-RT, delayed radiology reporting time

The most common disease category associated with radiologic misdiagnosis was digestive diseases (47.5%, 28 of 59). In those with D-RTs, neurologic diseases (46.8%, 29 of 62) were the most common disease category. Neurologic diseases (27.3%, 30 of 110) were also the most commonly associated category with clinician error, followed by infectious diseases (20.9%, 23 of 110) (Fig. 2; See Additional file 1-3 for a summary of the actual pathologic diseases associated with diagnostic errors and Additional file 1-4 for a summary of the causes and preventability of diagnostic errors).

### AO occurrence according to diagnostic error status and revisit timing

**Fig. 3:**
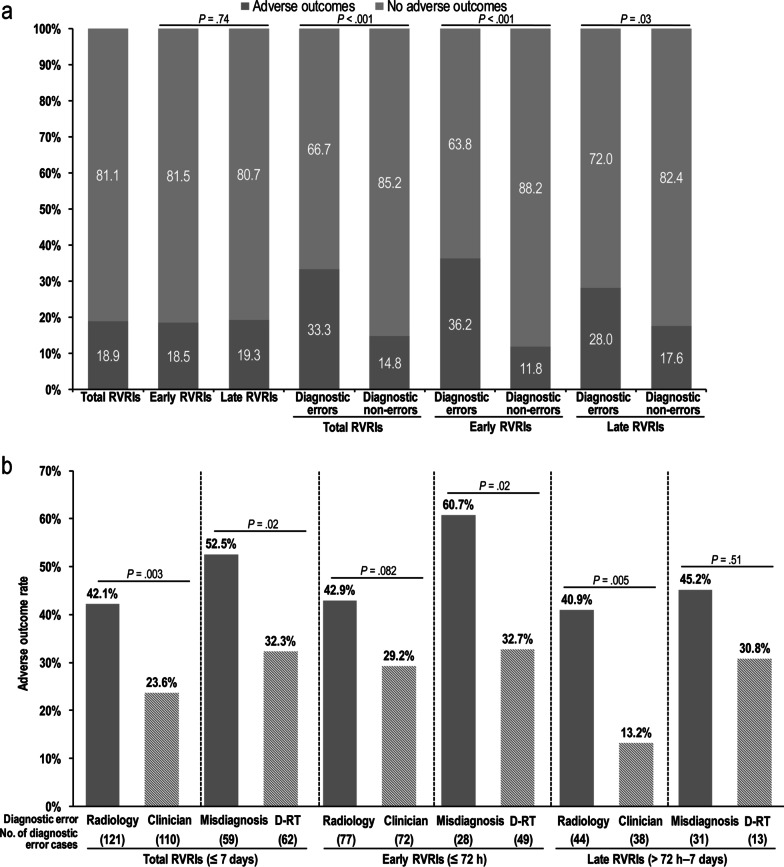
Adverse outcome rate among patients with emergency department revisits with repeat imaging (**a**) according to the presence or absence of diagnostic errors and revisit timing and (**b**) according to the type of diagnostic error and revisit timing. RVRIs, revisits with repeat imaging; Radiology, radiologic error; Clinician, clinician error; D-RT, delayed radiology reporting time

Figure 3 summarizes the AO rate according to the revisit timing and diagnostic error subtypes. The AO rate was the highest in patients with early revisits within 72 h if diagnostic errors occurred. The AO rate in the group with diagnostic errors was significantly higher than that in the non-error group, regardless of the revisit timing (early RVRIs, 36.2% [54 of 149] vs. 11.8% [47 of 397]; late RVRIs, 28.0% [23 of 82] vs. 17.6% [75 of 426]; all *p* < 0.05). The AO rate was significantly higher in the radiologic error group than in the clinician error group (42.1% [51 of 121] vs. 23.6% [26 of 110], *p* = 0.003) and higher in the misdiagnosis group than in the D-RT group (52.5% [31 of 59] vs. 32.3% [20 of 62], *p* = 0.02).

### Risk factors for AOs in association with RVRIs

Tables 2 and 3 summarize the risk factors for AOs. Multivariable logistic analyses revealed the following risk factors for AO occurrence in total RVRIs: radiologic misdiagnoses (odds ratio [OR] 6.75; *p* < 0.001), D-RT (OR 2.56; *p* = 0.002), clinician errors (OR 2.18; *p* = 0.005), digestive diseases (OR 2.81; *p* = 0.003), neoplasms (OR 5.67; *p* < 0.001), circulatory diseases (OR 6.13; *p* < 0.001), and healthcare-related complications (OR 5.31; *p* = 0.006). Radiologic misdiagnosis was a risk factor for AOs regardless of the revisit timing (early RVRIs, OR 14.43; *p* < 0.001 and late RVRIs, OR 4.02; *p* = 0.001). In early RVRIs, D-RT (OR 3.3; *p* = 0.001) and clinician error (OR 3.78; *p* < 0.001) were risk factors for AOs. In early RVRIs, diagnoses of neoplasms (OR 4.2; *p* = 0.004) and circulatory diseases (OR 6.32; *p* = 0.006) were risk factors for AOs. In late RVRIs, diagnoses of digestive diseases (OR 3.36; *p* = 0.02), neoplasms (OR 5.52; *p* < 0.001), respiratory diseases (OR 3.53; *p* = 0.04), circulatory diseases (OR 5.66; *p* = 0.02), and healthcare-related complications (OR 7.8; *p* = 0.04) were risk factors for AOs.

**Table 2:** Univariable logistic regression analysis of risk factors for adverse outcomes in revisits with repeat imaging

Variables	Total RVRIs		Early RVRIs		Late RVRIs	
	OR (95% CI)	*P*	OR (95% CI)	*P*	OR (95% CI)	*P*
Age	1.01 (1–1.02)	.05	1.01 (1–1.02)	.21	1.01 (1–1.03)	.14
Sex, male	1.33 (0.98–1.82)	.07	1.27 (0.82–1.96)	.28	1.4 (0.9–2.18)	.14
*Comorbidities*						
CAD	1.34 (0.81–2.22)	.26	1.08 (0.5–2.31)	.85	1.61 (0.82–3.16)	.17
Asthma	0.47 (0.14–1.56)	.22	0.48 (0.11–2.1)	.33	0.46 (0.06–3.67)	.46
COPD	2.06 (0.88–4.85)	.10	1.79 (0.55–5.84)	.33	2.45 (0.7–8.54)	.16
CKD	0.74 (0.34–1.59)	.44	0.62 (0.18–2.11)	.44	0.83 (0.31–2.22)	.71
DM	0.99 (0.67–1.46)	.95	1.31 (0.74–2.29)	.35	0.77 (0.44–1.33)	.34
Malignancy	2.03 (1.48–2.78)	< .001*	2.16 (1.36–3.43)	.001*	2.01 (1.28–3.14)	.002*
Revisit timing, late	1.05 (0.77–1.43)	.74				
*Diagnostic error status*						
Misdiagnoses	6.36 (3.69–10.98)	< .001	11.51 (5.08–26.06)	< .001*	3.85 (1.82–8.16)	< .001*
D-RT	2.74 (1.55–4.82)	< .001	3.61 (1.85–7.06)	< .001*	2.08 (0.62–6.93)	.23
Clinician errors	1.78 (1.1–2.87)	.02	3.07 (1.7–5.55)	< .001*	0.71 (0.27–1.88)	.49
Non-errors	1	< .001	1	< .001	1	.004
*Disease categories*						
1. Neurologic diseases	1.74 (0.92–3.29)	.09	1.67 (0.73–3.84)	.23	1.79 (0.67–4.81)	.25
2. Digestive diseases	4.14 (2.17–7.92)	< .001*	3.96 (1.63–9.63)	.002*	4.44 (1.71–11.57)	.002*
3. Neoplasms	5.41 (2.84–10.3)	< .001*	6.12 (2.46–15.23)	< .001*	5.3 (2.07–13.56)	< .001*
4. Traumatic injuries	0.68 (0.25–1.82)	.44	0.36 (0.07–1.74)	.20	1.2 (0.32–4.5)	.79
5. Respiratory diseases	2.62 (1.08–6.35)	.03*	1.7 (0.41–7.08)	.47	3.55 (1.08–11.65)	.04*
6. Genitourinary diseases	1.42 (0.48–4.19)	.53	1.01 (0.2–5.16)	.99	1.94 (0.44–8.54)	.38
7. Infections	1.59 (0.54–4.73)	.40	1.6 (0.39–6.65)	.52	1.56 (0.29–8.47)	.61
8. Circulatory diseases	8.53 (3.42–21.29)	< .001*	12.37 (3.63–42.21)	< .001*	5.19 (1.22–21.95)	.03*
9. Healthcare-related complications	5.73 (1.84–17.83)	.003*	4.81 (1.18–19.59)	.03*	7.78 (1.08–55.99)	.04*
10. Other diseases	1	< .001	1	< .001	1	.001

Data are shown as odds ratios with 95% confidence intervals in parentheses. Univariable logistic analysis was performed with backward elimination using penalized maximum likelihood estimation.

CAD, Coronary artery disease; CKD, chronic kidney disease; COPD, chronic obstructive pulmonary disease; DM, diabetes mellitus; ED, emergency department; RVRIs, revisits with repeat imaging; D-RT, delayed radiology reporting time; CI, confidence interval; OR, odds ratio

*p values < .05

**Table 3:** Multivariable logistic regression analysis of risk factors for adverse outcomes in revisits with repeat imaging

Variables	Total RVRIs		Early RVRIs		Late RVRIs	
	OR (95% CI)	*P*	OR (95% CI)	*P*	OR (95% CI)	*P*
Age	1.01 (1–1.02)	.02			1.02 (1–1.03)	.06
Comorbidity (malignancy)			1.83 (1.01–3.32)	.05		
*Diagnostic error status*						
Misdiagnoses	6.75 (3.67–12.4)	< .001*	14.43 (5.59–37.24)	< .001*	4.02 (1.75–9.25)	.001*
D-RT	2.56 (1.4–4.66)	.002*	3.3 (1.59–6.83)	.001*	1.93 (0.56–6.69)	.30
Clinician errors	2.18 (1.26–3.78)	.005*	3.78 (1.85–7.7)	< .001*	0.86 (0.3–2.46)	.78
Non-errors	1	< .001	1	< .001	1	.01
*Disease categories*						
1. Neurologic diseases	1.58 (0.83–3.04)	.17	1.32 (0.55–3.16)	.54	1.81 (0.67–4.92)	.24
2. Digestive diseases	2.81 (1.42–5.55)	.003*	2.04 (0.78–5.32)	.15	3.36 (1.25–9.07)	.02*
3. Neoplasms	5.67 (2.93–10.97)	< .001*	4.2 (1.57–11.23)	.004*	5.52 (2.13–14.29)	< .001*
4. Traumatic injuries	0.69 (0.25–1.87)	.46	0.37 (0.07–1.91)	.24	1.2 (0.32–4.54)	.79
5. Respiratory diseases	2.22 (0.9–5.5)	.08	1.08 (0.24–4.91)	.92	3.53 (1.06–11.8)	.04*
6. Genitourinary diseases	0.88 (0.28–2.74)	.83	0.36 (0.06–2.2)	.27	1.47 (0.32–6.7)	.62
7. Infections	0.99 (0.31–3.19)	.99	0.69 (0.15–3.2)	.63	1.4 (0.23–8.52)	.72
8. Circulatory diseases	6.13 (2.36–15.95)	< .001*	6.32 (1.69–23.62)	.006*	5.66 (1.31–24.44)	.02*
9. Healthcare-related complications	5.31 (1.63–17.27)	.006*	3.47 (0.75–16.01)	.11	7.8 (1.08–56.59)	.04*
10. Other diseases	1	< .001	1	< .001*	1	.002

Data are shown as odds ratios with 95% confidence intervals in parentheses. Multivariable logistic analysis was performed with backward elimination using penalized maximum likelihood estimation with variables for which statistically significant correlations were found in the univariable analysis

RVRIs, Revisits with repeat imaging; D-RT, delayed radiology reporting time; OR, odds ratio; CI, confidence interval

*p values < .05

After adjusting for potential confounding factors, the subtypes of imaging-related diagnostic errors differently contributed to the occurrence of AOs. In the matched set, the risk factors for AOs were radiologic error (OR 3.56; *p* < 0.001) in total RVRIs, radiologic error (OR 3.70; *p* = 0.001) and clinician error (OR 4.82; *p* = 0.03) in early RVRIs, and radiologic error (OR 3.36; *p* = 0.02) in late RVRIs (Table 4).

**Table 4:** Logistic regression analysis for 1:1 matching between patients with adverse outcomes and the control group

	No. of total patients (no. of adverse outcomes)		Odds ratio (95% CI)	*p*
	Study group	Matched control group		
*Total RVRIs*				
All diagnostic errors	195 (59)	195 (26)	2.82 (1.72–4.63)	< .001*
Radiologic errors	107 (43)	107 (17)	3.56 (1.90–6.66)	< .001*
Clinician errors	88 (16)	88 (9)	1.95 (0.82–4.62)	.13
*Early RVRIs* (≤ 72 h)				
All diagnostic errors	120 (38)	120 (13)	3.81 (1.94–7.50)	< .001*
Radiologic errors	64 (26)	64 (10)	3.70 (1.65–8.27)	.001*
Clinician errors	56 (12)	56 (3)	4.82 (1.20–19.43)	.03*
*Late RVRIs* (> 72 h–7 days)				
All diagnostic errors	75 (21)	75 (13)	1.86 (0.89–3.89)	.10
Radiologic errors	43 (17)	43 (7)	3.36 (1.24–9.14)	.02*
Clinician errors	32 (4)	32 (6)	0.62 (0.20–1.96)	.41

The two groups were matched for age (± 5 years), sex, revisit timing, comorbidity (malignancy), and six disease categories (neurologic, digestive, neoplasm, respiratory, circulatory, and other diseases). In the matched set, binary outcomes were compared using logit models with generalized estimating equations, which accounted for the clustering of matched pairs.

RVRIs, Revisits with repeat imaging; CI, confidence interval

*p values < .05.

## Discussion

Few studies have been published on diagnostic errors in the context of emergency imaging studies and their associations with AOs. To our knowledge, this is the first study to analyze this issue using RVRIs as a trigger. We found that the rate of AOs related to diagnostic errors increased up to 36.2% (54 of 149) in early RVRIs. This was much higher than the AO rate (11.8% [47 of 397]) in the non-diagnostic error group.

Our study identified diagnostic errors as the main risk factors for AOs. Interestingly, diagnostic errors were more strongly associated with the occurrence of AOs in early RVRIs than in late RVRIs. In terms of the investigated diagnostic error categories, radiologic misdiagnosis was the category most likely to be associated with AOs regardless of the revisit timing. D-RTs and clinician errors were risk factors for AOs mainly in association with early RVRIs. This might be because most patients who experienced D-RTs or clinician errors returned to the ED during the early period after discharge owing to urgent recalls for abnormal imaging findings or rapid disease progression. In contrast, patients with radiologic misdiagnoses at the index visits may differ in revisit timing according to disease severity. Our data imply that regardless of the revisit timing, emergency radiologists should consider the possibility of misdiagnosis stemming from index imaging among returning patients who require repeat imaging. Moreover, AOs associated with D-RTs during the early period could be prevented by improving the emergency radiology service. Our study underscore the importance of minimizing diagnostic errors in emergency imaging studies to reduce AOs and improve emergency care.

Previous related studies have investigated diagnostic errors with relatively long-term imaging-based follow-up results or have been based on selected medical malpractice claim cases [14–22]. Lee et al. [20] reported that repeat abdominal CT within 1 month yielded new or worse findings for 30% of ED patients with abdominal pain. Siegal et al. [21] reported on 1325 medical malpractice cases for which radiology was identified as the primarily responsible service. The diagnostic error rate was nearly 60%, and the leading cause of diagnostic error was misinterpretation (48%). However, most cases in that study occurred in ambulatory services (63%) and among inpatients (26%), which means that the results do not reflect diagnostic errors in the ED. Calder et al. [6] reported that 6.8% of patients returned to the ED within 7 days and that the AO rate was 12% among patients who returned to the ED within 72 h. These results were similar to ours. However, they focused only on clinician-associated issues (management, procedures, medication, and actions on laboratory and imaging findings) in a small number of patients. These studies are limited in reflecting ED diagnostic errors stemming from imaging studies and their association with AOs.

Besides diagnostic errors as risk factors for AOs, our study identified disease categories associated with diagnostic errors and AOs. Previous studies suggested disease categories associated with repeat imaging or those in medical malpractice claim cases [14, 20, 21, 31]. In agreement with previous studies, our study showed that neurologic diseases and neoplasms were frequent among cases associated with diagnostic errors. However, unlike neoplasms, neurologic diseases were not a risk factor for AOs in our study. This may be because traumatic subarachnoid hemorrhages and minor strokes or transient ischemic attacks predominated in this category, and these conditions have a low risk of rapid deterioration and do not need surgical intervention or aggressive management [32–34]. However, the clinical relevance of neurologic diseases to AOs must be carefully interpreted, as neurologic diseases are potentially destructive (e.g., recurrent stroke) [35] and frequently associated with diagnostic errors and AOs. Notably, in our study, digestive diseases were the most frequently misdiagnosed conditions by radiologists and were a significant risk factor for AOs in association with RVRIs. These results are supported by the findings of previous studies. Carrara et al. [36] showed that abdominal diseases accounted for the largest proportion (44.1%) of diagnostic errors stemming from CT and MRI interpretation. Chang et al. [37] reported that digestive diseases were common among patient revisits within 72 h in association with ED admissions to the ICU. In line with a previous study [37], our study showed that circulatory and respiratory diseases, which were frequently associated with clinician errors, were risk factors for AOs. Despite their low frequency, healthcare-related complications also contributed to the occurrence of AOs. An improved understanding of these disease categories in this context may help reduce the rates of diagnostic errors and AOs associated with RVRIs in the ED.

This study had several limitations. First, it was performed at a single tertiary training hospital. Quality care indicators heavily rely on the emergency care system, health insurance system, health costs, and variable resources. Therefore, our results may have limited generalizability. Second, the retrospective study design over a long period may limit the evaluation of certain details concerning clinical decision-making (e.g., communication errors, experience of radiologists, or disease severity). The emergency care process is difficult to define because of its complexity, and our analysis of diagnostic errors in the context of RVRIs may not fully reflect this complexity. Third, the long study period might have introduced unrecognized biases due to emergency care and radiology service improvements over time. For example, in the last decade, D-RTs have decreased from a few days to 30 min in many institutions [38]. However, D-RTs are still important quality control factors in many institutions, and further D-RT improvements in the emergency setting would be beneficial. Fourth, the incidence of repeat imaging and diagnostic errors in this study may have been underestimated owing to cases of loss to follow-up and transfer to other hospitals.

## Conclusions

Our study suggests that diagnostic errors stemming from emergency imaging studies are strongly associated with AOs in patients with RVRIs in the ED. In the present study, neurologic and digestive diseases were the most common medical conditions associated with diagnostic errors. Our results also showed that the AO rate was the highest in early revisits within 72 h if diagnostic errors occurred. These findings underscore the importance of paying special attention to early revisitors with suspected neurologic or digestive diseases. The index CT could provide valuable clues to the accurate triage of revisiting patients before clinicians order repeat examinations. In addition, our data suggest that RVRIs could be a good quality indicator for emergency radiology services. Strategies such as reviewing RVRIs, the AOs of patients, and the error documentations may reduce the diagnostic error rate over time. The present study provides valuable information for the establishment of strategies for minimizing diagnostic errors and improving emergency radiology services.

## Supplementary Information

**Additional file 1**. **1-1** The emergency radiology service system. **1-2** The study design and classification criteria of diagnostic errors in the index imaging studies. **1-3** The actual pathologic diseases associated with diagnostic errors in the index imaging studies. **1-4** The analysis of the causes and preventability of diagnostic errors.

## Data Availability

The datasets used and/or analyzed during the current study are available from the corresponding author on reasonable request.

## Abbreviations

AOs: Adverse outcomes
CI: Confidence interval
D-RT: Delayed radiology reporting time
ED: Emergency department
ICU: Intensive care unit
OR: Odds ratio
RVRIs: Revisits with repeat imaging
