# Supplementary material for: Impact of diagnostic errors on adverse outcomes: learning from emergency department revisits with repeat CT or MRI
Source: Insights Imaging. 2021 Nov 3;12:160. doi: 10.1186/s13244-021-01108-0 (PMC8566620; doi:10.1186/s13244-021-01108-0)
Supplement: Supplementary file 1 — Additional file 1. 1-1 The emergency radiology service system. 1-2 The study design and classification criteria of diagnostic errors in the index imaging studies. 1-3 The actual pathologic diseases associated with diagnostic errors in the index imaging studies. 1-4 The analysis of the causes and preventability of diagnostic errors. [file 13244_2021_1108_MOESM1_ESM.docx]

**ELECTRONIC SUPPLEMENTARY MATERIAL**

**Emergency radiology service system, detailed study design, diagnostic error-related disease list, and causes and preventability of diagnostic errors**

***Additional file 1-1:***

**The emergency radiology service system**

Due to the long study period, the present study included various emergency radiology service systems in our institution. Before 2011, staff radiologists with different subspecialties and residents provided emergency department (ED) coverage during daytime hours (8:00 am to 5:00 pm on weekdays). Outside regular working hours (5:00 pm to 8:00 am on weekdays and a day on weekends), senior radiology residents provided preliminary reports to the referring clinicians at the ED while on call. According to the imaging studies, seven staff radiologists with subspecialties (abdominal, chest, cardiovascular, neuro-, pediatric, genitourinary, musculoskeletal radiologists) were available on call. Staff radiologists then reviewed the preliminary reports and signed off the final reports the following day. In January 2011, our institution began to provide an in-house emergency radiology staff service. The dedicated emergency radiology section was established as an integral part of this service. This task force consisted of one ED-dedicated staff radiologist and four ED-non-dedicated staff radiologists (two abdominal radiologists, one chest radiologist, and one neuroradiologist). Because the study volumes at the ED were highest in the evening, in-house staff radiologists were assigned in the evening (5:00 pm to 10:00 pm on the weekdays) and during the day on weekends and holidays (from 1:00 pm to 9:00 pm). In March 2014, another ED-dedicated radiology staff member was recruited in the dedicated emergency radiology section. From 2016 to 2018, two ED-dedicated staff radiologists alternatively covered the expanded working hours (8:00 am to 10:00 pm on weekdays and 1:00 pm to 9:00 pm on weekends and holidays). Since 2019, three in-house ED-dedicated radiologists have been rostered on, covering more working hours (8:00 am to 10:00 pm on weekdays and 9:00 am to 9:00 pm on weekends and holidays).

***Additional file 1-2:***

**The study design and classification criteria of diagnostic errors in the index imaging studies**

We analyzed the nature of patient revisits with repeat imaging (RVRIs) according to a modified classification with reference to a previous study [1]. The nature of RVRIs was categorized into three groups: 1) radiologic error (misdiagnosis or delayed radiology reporting time [D-RT]), 2) clinician error (inappropriate patient triage or failure to order appropriate imaging examinations), and 3) non-error (patient- or illness-related factor: progressed diseases or new symptoms or diseases). The details of each category are summarized in Table S1. In addition to radiologic errors, the present study analyzed clinician errors in ordering imaging examinations. This is because previous studies identified clinician error as one of the major causes of unnecessary requests for repeat tests [2-3]. Our study did not include patient revisits without repeat imaging, because there is no reference standard for critical findings on early-stage diseases. Although two board-certified radiologists (GS Hong and KJ Park) reviewed the images in consensus, the analysis of diagnostic errors in early-stage diseases could be subjective in the cases without reference imaging (i.e., repeat imaging). Thereby, it could lead to a selection bias by researchers. To mitigate selection bias, our study was performed only in RVRIs.

For the analysis of misdiagnosis in radiology, three radiologists (GS Hong, KJ Park, and Y Ahn, with > 14, > 8, and 4 years of experience in CT and MRI, respectively) reviewed the radiology reports of index and repeat imaging studies. When there were discrepancies on critical findings between two radiology reports, two board-certified radiologists (GS Hong and KJ Park) reviewed the images to identify whether or not the discrepancies were due to a misdiagnosis. The consensus about radiologic errors was reached through a discussion. When the radiology reporting time was later than the clinician’s decision time, two radiologists (GS Hong and KJ Park) reviewed the index images and considered cases with positive critical findings as D-RT. The reporting time in preliminary written reports was used as a reference in determining D-RT because preliminary reporting is commonly used in making clinical decisions in the emergency setting. The reporting time in the final reports was also used in cases with a discordance on critical findings between the preliminary and final radiology reports. In these cases, failure to report timely in the final report was also considered a D-RT. Clinician errors comprised inappropriate patient triage (i.e., underestimation of the patient’s condition and scanning at inappropriate sites) and failure to order appropriate imaging examinations (i.e., the ordered imaging studies were unsuitable for diagnosing the patient’s disease). However, detecting positive findings on an index scan regardless of clinician error (e.g., unreported acute cholecystitis present on the index chest CT scan) was considered a radiologic misdiagnosis.

**Table S1.** Classification of diagnostic error status at the index imaging in the emergency department

|  |  | Definition | Example |
| --- | --- | --- | --- |
| Radiologic error | Misdiagnosis | Misdiagnosis is defined as an incorrect diagnosis of critical findings on index images by reference to repeat images. A critical finding is defined as any radiologic finding on index images relevant to the patient’s symptoms, which has an immediate impact on patient care and is of an emergency or urgent nature. This error is based on the final written report. | Dural sinus venous thrombosis was present on the index image but was not noticed at the time of the index image analysis. |
|  | D-RT | Delayed radiology reporting time is defined as cases in which the radiologic reports about critical findings on index images were made after the clinical decision on the patient’s treatment or disposition. This category does not cover preliminary verbal reports owing to the retrospective study design. The reporting time in the final reports was also used in cases with a discordance on critical findings between the preliminary and final radiology reports. In these cases, failure to report timely in the final report was also considered a D-RT. | A small amount of traumatic subarachnoid hemorrhage was present on the index image but was not timely reported by radiologists. |
| Clinician error | Inappropriate patient triage or failure to order appropriate imaging examinations | Inappropriate patient triage is defined as when the condition of the patient is underestimated, resulting in the patient not undergoing imaging examinations at the necessary sites. Failure to order appropriate imaging examinations is defined as situations in which the patient undergoes CT or MRI in the necessary scanning sites but the ordered examinations were not suitable for diagnosing the patient’s disease. This category does not include cases in which the condition of the patient is overestimated and the patient undergoes imaging examinations at multiple sites. This is because multifocal imaging examinations are not unusual in older patients or patients presenting with systemic symptoms in the emergency department [4]. | Inappropriate patient triage:  - In the index visit, an ED clinician ordered only CT coronary angiography for a patient with epigastric pain. The patient revisited the ED and underwent abdominopelvic CT. The patient was finally diagnosed with acute cholecystitis.  Failure to order appropriate imaging examinations:  - Initially, an ED clinician ordered cervical spine CT for a patient who presented with neck pain without a trauma history. In the revisit, the patient underwent CT neck angiography and was diagnosed with vertebral artery dissection. |
| Non-error | Patient- or illness-related factor | Patient factor includes disease progression or recurrence, development of a new disease, or unexpected medical complications, in the absence of problems with the diagnostic process in the index visit. This category does not include inadequate treatment in the index visit. | If a patient with abdominal pain was diagnosed with cancer progression at the index visit and revisited the same ED because of a traffic accident, it was considered a non-error. |

D-RT, delayed radiology reporting time; ED, emergency department; CT, computed tomography; MRI, magnetic resonance imaging.

**References**

1. Wu C-L, Wang F-T, Chiang Y-C et al (2010) Unplanned emergency department revisits within 72 hours to a secondary teaching referral hospital in Taiwan. J Emerg Med 38:512-517

2. Miyakis S, Karamanof G, Liontos M, and Mountokalakis TD (2006) Factors contributing to inappropriate ordering of tests in an academic medical department and the effect of an educational feedback strategy. Postgrad Med J 82: 823–829.

3. Gandhi TK, Kachalia A, Thomas EJ et al (2006) Missed and delayed diagnoses in the ambulatory setting: a study of closed malpractice claims. Ann Intern Med 145:488-496.

4. Moon S-H, Shim JL, Park K-S, Park C-S (2019) Triage accuracy and causes of mistriage using the Korean Triage and Acuity Scale. PLoS One 14:e0216972

***Additional file 1-3:***

**The** **actual pathologic diseases associated with diagnostic errors in the index imaging studies**

Our study showed that although most cases (78%) of patient revisits with repeat imaging (RVRIs) were due to non-errors (the progression of existing diseases or manifestation of a new disease), diagnostic errors were a contributing factor to lead to RVRIs. Interestingly, despite their smaller proportion, the adverse outcome rate in the diagnostic error group compared to the non-error group was significantly higher (33.3% vs. 14.8%, *P* < .001). Thus, it is crucial to know cases associated with such diagnostic errors. Table S2 summarizes the actual pathologic diseases associated with the diagnostic errors in the index imaging studies. It does not include the cases with RVRIs due to the progression of existing diseases or manifestation of a new disease. Digestive disease was the most frequently misdiagnosed condition by radiologists (47.5%, 28 of 59), and neurologic disease was the most common disease category associated with D-RT (46.8%, 29 of 62). Specifically, acute cholecystitis was the most frequently misdiagnosed disease (20.3%, 12 of 59) and intracranial hemorrhage was the condition most commonly associated with delayed radiologic reporting (30.6%, 19 of 62). Neurologic disease was the most common disease overlooked by ED physicians (27.3%, 30 of 110). Overall, the most common disease associated with clinician errors was meningitis (13.6%, 15 of 110), followed by pneumonia (9.1%, 10 of 110), spontaneous intracranial hypotension (9.1%, 10 of 110), and Guillain–Barré syndrome (4.5%, 5 of 110).

**Table S2.** List of actual pathologic diseases associated with diagnostic errors in the index imaging studies

| **Final diagnoses of diseases** | **All diagnostic errors**  **(n = 231)** | **Radiologic errors**  **(n = 121)** | | **Clinician errors**  **(n = 110)** |
| --- | --- | --- | --- | --- |
|  |  | **Misdiagnoses**  **(n = 59)** | **D-RT**  **(n = 62)** |  |
| **Neurologic diseases** | 67 (20) | 8 (3) | 29 (7) | 30 (10) |
| Intracranial hemorrhage | 23 (7) | 3 (2) | 19 (5) | 1 |
| Cerebral ischemia | 16 (3) | 3 | 8 (2) | 5 (1) |
| Spontaneous intracranial hypotension | 10 (3) |  |  | 10 (3) |
| Guillain–Barré syndrome | 5 (2) |  |  | 5 (2) |
| Cerebral venous thrombosis | 2 | 1 | 1 |  |
| Encephalitis | 2 (1) |  |  | 2 (1) |
| Cerebral aneurysm | 1 (1) |  |  | 1 (1) |
| RCVS | 1 |  |  | 1 |
| Spinal arteriovenous malformation | 1 |  |  | 1 |
| Carotid cavernous fistula | 1 (1) | 1 (1) |  |  |
| Myelitis | 1 |  |  | 1 |
| Methotrexate toxicity | 1 |  | 1 |  |
| Dissection of cerebral arteries | 1 (1) |  |  | 1 (1) |
| Dissection of intracranial vertebral artery | 2 (1) |  |  | 2 (1) |
| **Digestive diseases** | 52 (28) | 28 (18) | 13 (6) | 11 (4) |
| Acute cholecystitis | 18 (14) | 12 (10) | 3 (1) | 3 (3) |
| Bowel perforation | 7 (5) | 2 (2) | 5 (3) |  |
| Acute appendicitis | 4 (3) | 2 (2) | 1 (1) | 1 |
| Acute pancreatitis | 1 (1) | 1 (1) |  |  |
| Enterocolitis | 3 |  |  | 3 |
| Cholangitis | 3 | 2 |  | 1 |
| Common bile duct stone | 3 (1) | 2 | 1 (1) |  |
| Liver abscess | 3 (1) | 1 | 1 | 1 (1) |
| Hepatitis | 2 |  |  | 2 |
| Bowel ischemia | 2 (1) | 1 (1) | 1 |  |
| Bowel obstruction | 2 (1) | 1 (1) | 1 |  |
| Spontaneous bacterial peritonitis | 2 | 2 |  |  |
| Varix of the small intestine | 1 | 1 |  |  |
| Diverticulitis | 1 (1) | 1 (1) |  |  |
| **Infections** | 27 (2) | 4 (2) | 0 | 23 |
| Meningitis | 15 |  |  | 15 |
| Scrub typhus | 3 |  |  | 3 |
| Tuberculosis lymphadenopathy | 1 |  |  | 1 |
| Invasive fungal sinusitis | 1 (1) | 1 (1) |  |  |
| Malaria | 2 |  |  | 2 |
| Typhoid fever | 1 |  |  | 1 |
| Cellulitis | 1 |  |  | 1 |
| Fournier gangrene | 1 (1) | 1 (1) |  |  |
| Operative site infection | 2 | 2 |  |  |
| **Neoplasms** | 16 (8) | 7 (4) | 6 (4) | 3 |
| Leptomeningeal metastasis | 6 (2) | 1 | 2 (2) | 3 |
| Brain tumor | 4 (1) | 1 | 3 (1) |  |
| Hepatocellular carcinoma | 2 (2) | 2 (2) |  |  |
| Lymphoma/leukemia | 2 (1) | 1 | 1 (1) |  |
| Gastric cancer | 1 (1) | 1 (1) |  |  |
| Ovarian tumor | 1 (1) | 1 (1) |  |  |
| **Respiratory diseases** | 15 (2) | 0 | 3 (1) | 12 (1) |
| Pneumonia | 10 (1) |  |  | 10 (1) |
| Acute sinusitis | 1 (1) |  | 1 (1) |  |
| Acute tonsillitis | 1 |  |  | 1 |
| Influenza | 1 |  |  | 1 |
| Mediastinitis | 1 |  | 1 |  |
| Pneumothorax | 1 |  | 1 |  |
| **Circulatory diseases** | 15 (7) | 0 | 4 | 11 (7) |
| Pulmonary thromboembolism | 6 (3) |  | 3 | 3 (3) |
| Ischemic heart disease | 4 (2) |  |  | 4 (2) |
| Infectious endocarditis | 2 (1) |  |  | 2 (1) |
| Aortic dissection | 1 (1) |  |  | 1 (1) |
| Superior mesenteric artery dissection | 1 |  | 1 |  |
| Mitral valve prolapse | 1 |  |  | 1 |
| **Genitourinary diseases** | 15 (3) | 7 (3) | 0 | 8 |
| Acute pyelonephritis | 6 | 2 |  | 4 |
| Urinary tract infection | 3 |  |  | 3 |
| Bladder rupture | 2 (2) | 2 (2) |  |  |
| Pelvic inflammatory disease | 1 | 1 |  |  |
| Infected hydrocele | 1 |  |  | 1 |
| Ovarian torsion | 1 (1) | 1 (1) |  |  |
| Renal arteriovenous malformation | 1 | 1 |  |  |
| **Traumatic injuries** | 9 (3) | 2 | 3 | 4 (3) |
| Skull or facial bone fracture | 3 | 1 | 2 |  |
| Rib fracture | 1 | 1 |  |  |
| Compression fracture | 1 (1) |  |  | 1 (1) |
| First cervical vertebra fracture | 1 |  | 1 |  |
| Other fracture | 3 (2) |  |  | 3 (2) |
| **Healthcare-related complications** | 3 (1) | 1 | 2 (1) | 0 |
| Aorta anastomotic site leakage | 1 (1) |  | 1 (1) |  |
| Postoperative bile leakage | 2 | 1 | 1 |  |
| **Musculoskeletal or connective tissue diseases** | 5 (2) | 0 | 1 (1) | 4 (1) |
| Infectious spondyloarthritis | 3 (1) |  |  | 3 (1) |
| Skull base osteomyelitis | 1 (1) |  | 1 (1) |  |
| Adult-onset Still disease | 1 |  |  | 1 |
| **Visual system and ear diseases** | 6 (1) | 2 (1) | 1 | 3 |
| Orbital cellulitis/abscess | 4 (1) | 1 (1) | 1 | 2 |
| Inflammatory pseudotumor of the orbit | 1 | 1 |  |  |
| BPPV | 1 |  |  | 1 |
| **Endocrine diseases** | 1 | 0 | 0 | 1 |
| Hyponatremia | 1 |  |  | 1 |
| **Total** | 231 (77) | 59 (31) | 62 (20) | 110 (26) |

Note: Data are number of patients. Numbers in parentheses are the number of adverse outcomes applicable to each item. The diagnoses at the index visits were classified according to the modified guidelines from the 11th edition of the International Classification of Diseases. BPPV, benign paroxysmal positional vertigo; RCVS, reversible cerebral vasoconstriction syndrome; D-RT, delayed radiology reporting time; ED, emergency department

***Additional file 1-4:***

**The analysis of the causes and preventability of diagnostic errors**

The causes of radiologic errors were analyzed according to the modified categorization system with reference to a previous study [1] (Table S3). This modified categorization system considered delayed radiology reporting time (D-RT) as poor communication and excluded complications due to a procedure. In the RVRI, the most common cause of radiologic errors was poor communication (51.2%, 62 of 121), followed by perception (24.8%, 30 of 121) and cognitive errors (14.0%, 17 of 121). These results emphasize the importance of the timely recognition and reporting of emergency radiology examinations. In agreement with previous literature [2], our study shows that the perception error rate is higher than the cognitive error rate. According to the previous literature, preventable errors were considered to have occurred if a correct or timely diagnosis could have been readily expected with the given images and information, or something different could have been done to make the correct diagnosis earlier [3-5]. Preventability was analyzed in relation to the causes of diagnostic errors. The diagnostic errors were categorized by preventability as not preventable, possibly preventable, or definite preventable (Table S4). The overall preventable diagnostic error rate was 89.3% (108 of 121), in which the definite preventable diagnostic error rate was 78.5% (95 of 121), and the possibly preventable diagnostic error rate was 10.7% (13 of 121).

**Table S3.** Categorization of the causes of diagnostic errors

| **Type** | **Cause of error** | **Explanation** | **Error rate** |
| --- | --- | --- | --- |
| 1 | Complacency (Cognitive error) | Error of overreading and misinterpretation, in which a finding is appreciated but is attributed to the wrong cause | 2.5% (3) |
| 2 | Faulty reasoning (Cognitive error) | Error of overreading and misinterpretation, in which a finding is identified and interpreted as abnormal but is attributed to the wrong cause. Misleading information and a limited differential diagnosis are included in this category | 11.6% (14) |
| 3 | Lack of knowledge | The finding is seen but is attributed to the wrong cause because of a lack of knowledge on the part of the viewer or interpreter | 0.8% (1) |
| 4 | Underreading (Perception error) | The finding is missed | 24.8% (30) |
| 5 | Poor communication (Delayed radiology reporting time) | The lesion is identified and interpreted correctly, but the message fails to reach the clinician | 51.2% (62) |
| 6 | Technique | The finding is missed because of the limitations of the examination or technique | 3.3% (4) |
| 7 | Prior examination | The finding is missed because of failure to consult prior to radiologic studies or reports | 0.8% (1) |
| 8 | History | The finding is missed because of the acquisition of inaccurate or incomplete clinical history | 0.0% (0) |
| 9 | Location | The finding is missed because of the location of a lesion outside the area of interest on an image, such as in the corner of an image | 3.3% (4) |
| 10 | Satisfaction of search | The finding is missed because of failure to continue to search for additional abnormalities after the first abnormality was found | 1.7% (2) |
| 11 | Satisfaction of report | The finding was missed due to complacency and overreliance on the radiology report of the previous examinations | 0.0% (0) |

Note: Data are presented as a percentage (n). This categorization system is a modification of the classification schemes proposed by Kim and Mansfield [1].

**Table S4.** Categorization of the preventability of diagnostic errors

| **Category** | **Explanation** | **Relevant causes of diagnostic errors** |
| --- | --- | --- |
| Not preventable | The errors were due to limitations of the examination or technique, or the possibility of error always exists even though all preventable actions have been taken. | Cognitive error  Technique |
| Possible preventable | Making an accurate diagnosis could be possible if something different could be done or if additional clinical information was given. | Cognitive error |
| Definite preventable | Accurate diagnosis is expected or readily apparent given the images and information. | Lack of knowledge  Underreading  Poor communication  Prior examination  Location  Satisfaction of search  Satisfaction of report |

**References**

1. Kim YW, Mansfield LT (2014) Fool me twice: delayed diagnoses in radiology with emphasis on perpetuated errors. AJR Am J Roentgenol 202:465-470.

2. Bruno MA, Walker EA, Abujudeh HH (2015) Understanding and confronting our mistakes: the epidemiology of error in radiology and strategies for error reduction. Radiographics 35:1668-1676.

3. Singh H, Schiff GD, Graber ML, Onakpoya I, Thompson MJ (2017) The global burden of diagnostic errors in primary care. BMJ Qual Saf 26:484-494.

4. Brady AP (2017) Error and discrepancy in radiology: inevitable or avoidable? Insights Imaging 8:171-182.

5. Kruskal JB, Siewert B, Anderson SW, Eisenberg RL, Sosna J (2008) Managing an acute adverse event in a radiology department. Radiographics 28:1237-1250.
